# Antibacterial scalarane from *Doriprismatica stellata* nudibranchs (Gastropoda, Nudibranchia), egg ribbons, and their dietary sponge *Spongia* cf. *agaricina* (Demospongiae, Dictyoceratida)

**DOI:** 10.3762/bjoc.16.132

**Published:** 2020-07-03

**Authors:** Cora Hertzer, Stefan Kehraus, Nils Böhringer, Fontje Kaligis, Robert Bara, Dirk Erpenbeck, Gert Wörheide, Till F Schäberle, Heike Wägele, Gabriele M König

**Affiliations:** 1 Institute for Pharmaceutical Biology, University of Bonn, Nussallee 6, 53115 Bonn, Germany; 2 Institute for Insect Biotechnology, Justus-Liebig-University, Heinrich-Buff-Ring 26–32, 35392 Gießen, Germany; 3 Department for Bioresources of the Fraunhofer Institute for Molecular Biology and Applied Ecology, Ohlebergsweg 12, 35392 Gießen, Germany; 4 Faculty of Fisheries and Marine Science, Sam Ratulangi University, Jl. Kampus UNSRAT Bahu, 95115 Manado, Sulawesi Utara, Indonesia; 5 Department of Earth and Environmental Sciences, Palaeontology & Geobiology, Ludwig-Maximilians-Universität München, Richard-Wagner-Str. 10, 80333 München, Germany; 6 GeoBio-Center, Ludwig-Maximilians-Universität München, Richard-Wagner-Str. 10, 80333 München, Germany; 7 SNSB – Bayerische Staatssammlung für Paläontologie und Geologie, Richard-Wagner-Str. 10, 80333 München, Germany; 8 Zoologisches Forschungsmuseum Alexander Koenig, Adenauerallee 160, 53113 Bonn, Germany

**Keywords:** antibacterial, Dictyoceratida, Nudibranchia, scalarane, sesterterpene

## Abstract

Investigations on the biochemical relationship between *Doriprismatica stellata* (Chromodorididae, Doridoidea) nudibranchs, their egg ribbons, and the associated dietary sponge *Spongia* cf. *agaricina* (Demospongiae, Porifera) led to the isolation of the structurally new scalarane-type sesterterpene 12-deacetoxy-4-demethyl-11,24-diacetoxy-3,4-methylenedeoxoscalarin, with an unprecedented position of the cyclopropane ring annelated to the ring A. Unlike other scalaranes, which are most often functionalized at C-12 of ring C, it bears two acetoxy groups at C-11 and C-24 instead. The compound was present in all three samples, supporting the dietary relationship between chromodorid nudibranchs of the genus *Doriprismatica* and scalarane-containing dictyoceratid sponges of the Spongiidae family. The results also indicate that *D. stellata* passes the scalarane metabolite on to its egg ribbons, most likely for protective purposes. The scalarane showed antibacterial activity against the Gram-positive bacteria *Arthrobacter crystallopoietes* (DSM 20117) and *Bacillus megaterium* (DSM 32).

## Introduction

In habitats with intense competition and feeding pressure, such as coral reefs, sessile or slow-moving organisms commonly defend themselves with toxic or deterrent molecules [1–8]. Sponges (Porifera), for example, represent one of the main sources of marine bioactive natural products, due to their impressive chemical armoury [4]. These specialized metabolites can be produced either by the sponge itself or by associated microbial symbionts [9–16]. Their production is assumed to be useful against numerous environmental stress factors, such as predation, pathogens, overgrowth by fouling organisms, or competition for space [4,10,15,17].

Though defensive metabolites are effective against most predators, some also attract nudibranchs of the family Chromodorididae (Gastropoda, Mollusca). These colorful, shell-less sea slugs are specialized to live and feed on noxious demosponges (Demospongiae, Porifera). They evolved the ability to sequester, accumulate, and store spongian metabolites to their own advantage [2,5,9,18–33]. Besides, specific metabolites can be passed on from the sea slugs to their similarly conspicuous and physically defenceless eggs. This has been shown exemplarily for the egg ribbons of certain nudipleuran taxa, such as *Hexabranchus sanguineus* [17], *Pleurobranchaea maculata* [34], *Cadlina luteomarginata* [35], and the two *Dendrodoris* species *D. grandiflora* and *D. limbata* [36]. The passing on of special metabolites from sea slugs to their egg ribbons suggests an additional biological role in the reproductive cycle or as protection of the eggs against predation or fouling.

Chemotaxonomic approaches have shown that chromodorid nudibranchs of the genera *Chromodoris*, *Doriprismatica*, *Felimare*, *Felimida*, *Glossodoris/Casella*, and *Goniobranchus* sequester and reuse spongian-type furanoterpenoids, diterpenoids, and sesquiterpenoids, or scalarane-type sesquiterpenoids and sesterterpenoids from their sponge prey [23,37–45]. However, confusion in the chemotaxonomy of Chromodorididae arose by multiple changes in the species names, including splitting and synonymizations, and the inclusion of species that have since been discovered to be members of other genera. Additionally, a splitting of generic groups into several genera and resurrection of old names increased the confusion [39,42,46–49]. To classify specialized metabolites in the Chromodorididae in a meaningful way, a solid understanding of their taxonomy, biology, and prey is essential.

Members of *Glossodoris/Casella* and *Doriprismatica* represent such a case of complex systematic challenges and complicated taxonomic histories [49]. Previous work on *Doriprismatica* (former *Glossodoris*) *sedna* [39] and *Doriprismatica* (former *Glossodoris* or *Casella*) *atromarginata* [38,41,44–45,50], reported the isolation of scalaranes, homoscalaranes, norscalaranes, spongian diterpenoids and furanoditerpenoids. A dietary origin of these molecules was inferred and attributed to dictyoceratid sponges of the genera *Hyrtios* and *Carteriospongia* (Thorectidae), as well as *Hyattella* and *Spongia* (Spongiidae). A geographical variation was described between *D. atromarginata* populations from Sri Lanka and Australia, containing furanoditerpenes, and a *D. atromarginata* population from India, containing scalarane sesterterpenes as a consequence of sponge prey availability [41]. The isolated metabolites showed various biological activities, such as cytotoxicity, antimicrobial, antiviral and antitumor activities, inhibition of transactivation for the farnesoid X receptor, inhibition of mammalian phospholipase A_2_, and ichthyotoxicity against the mosquitofish *Gambusia affinis* [28–29,39,51–56]. Furthermore, a Vietnamese collection of *D. atromarginata* was found on the gorgonian *Menella woodin* (Plexauridae, Alcyonacea). Instead of spongian- or scalarane-type metabolites, they contained steroidal compounds, presumably sequestered from *M. woodin* [57].

**Figure 1:**
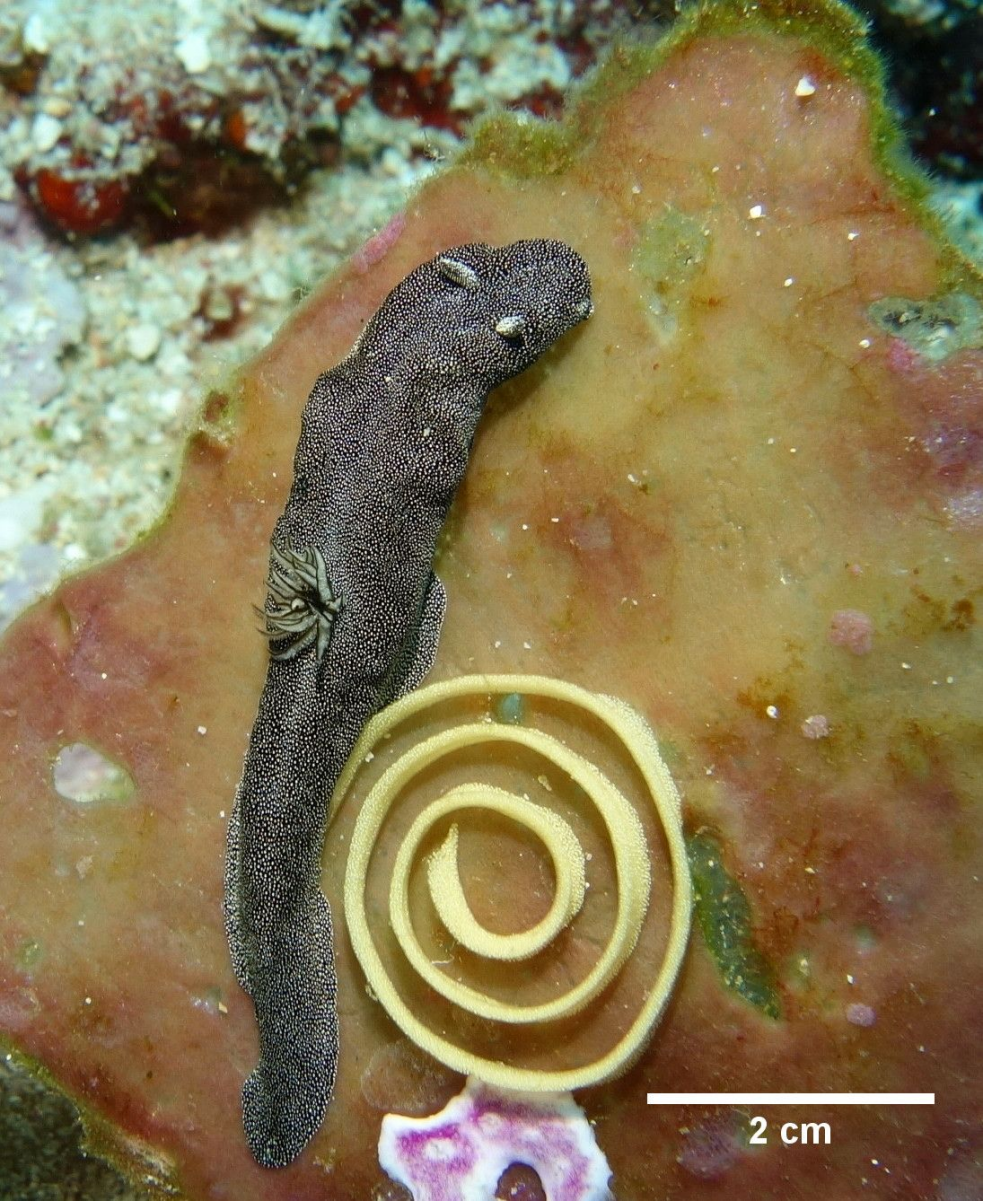
*Doriprismatica stellata* nudibranch, egg ribbon, and *Spongia* cf. *agaricina* specimen.

**Figure 2:**
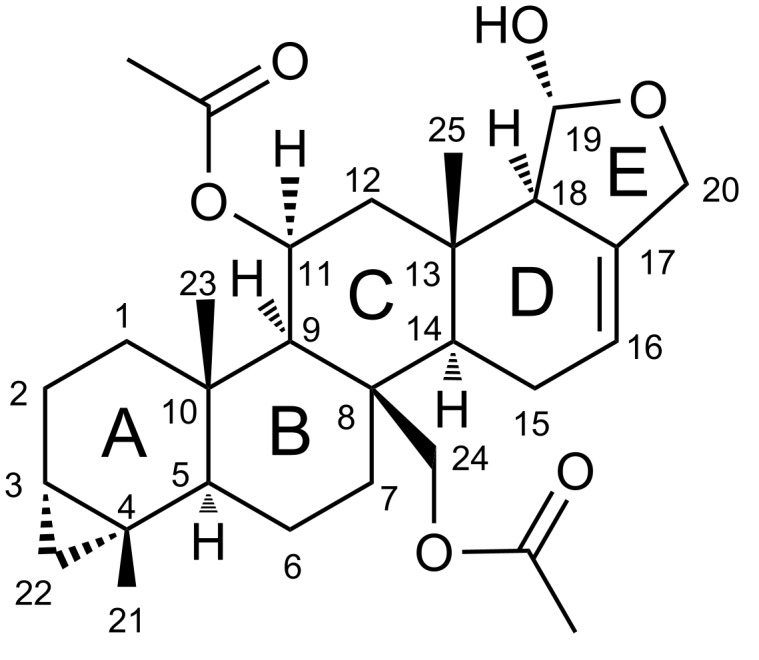
The structurally new 12-deacetoxy-4-demethyl-11,24-diacetoxy-3,4-methylenedeoxoscalarin (relative stereochemistry depicted), isolated from *Doriprismatica stellata* nudibranchs, their egg ribbons and the dietary sponge *Spongia* cf. *agaricina*.

**Figure 3:**
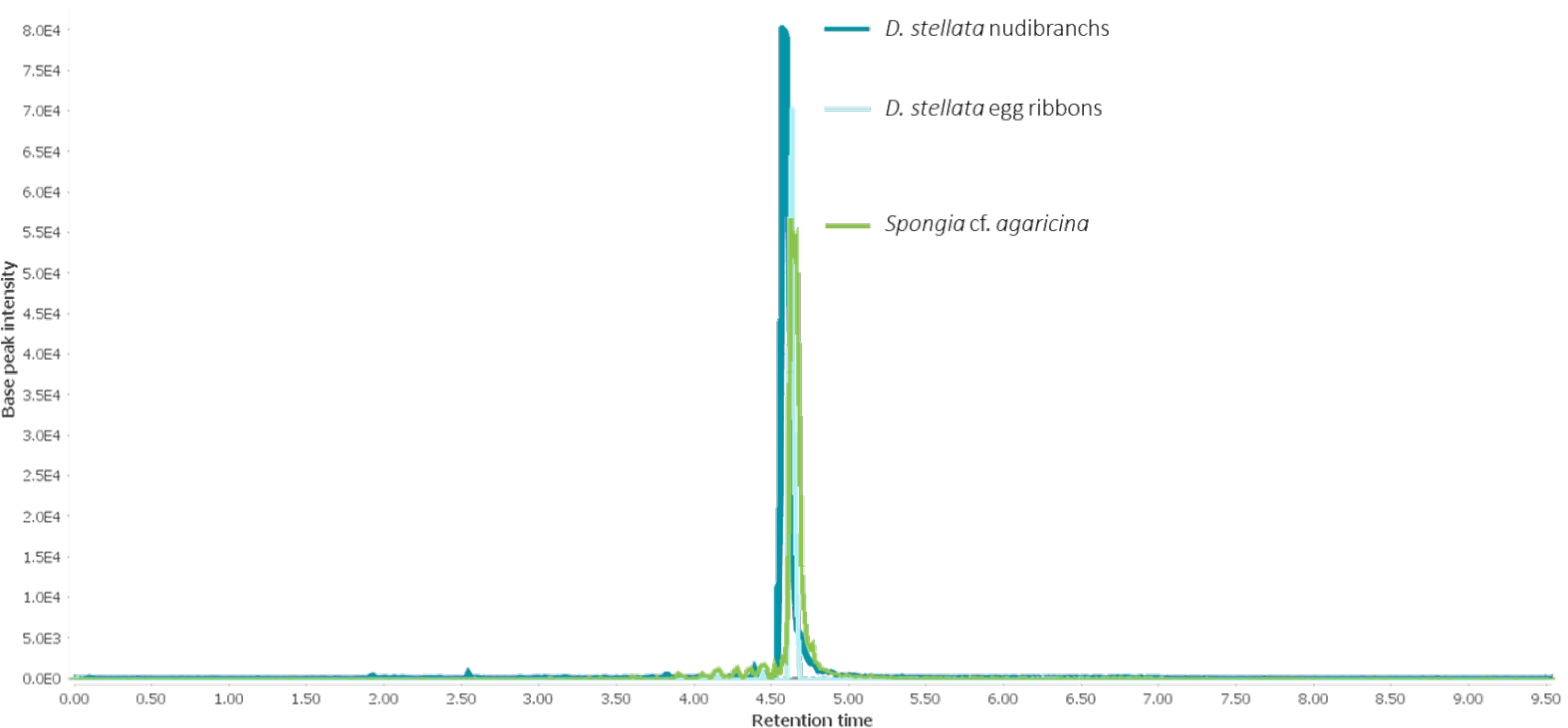
Superimposed HPLC–MS chromatogram of *Doriprismatica stellata* nudibranch, egg ribbon, and *Spongia* cf. *agaricina* extracts, showing the presence of 12-deacetoxy-4-demethyl-11,24-diacetoxy-3,4-methylenedeoxoscalarin in all three samples.

Here, we report the first investigation on the biochemical relationship between *Doriprismatica* (former *Glossodoris*) *stellata* (Chromodorididae, Doridina) of the Indo-West Pacific (Figure 1), their egg ribbons, and the associated dietary sponge, identified as *Spongia* cf. *agaricina* (Spongiidae, Demospongiae). We describe the structure elucidation of the new scalarane sesterterpene 12-deacetoxy-4-demethyl-11,24-diacetoxy-3,4-methylenedeoxoscalarin (Figure 2), isolated from all our *Doriprismatica stellata* nudibranch, egg ribbon and *Spongia* cf. *agaricina* samples (Figure 3). It is the first scalarane sesterterpene reported with a cyclopropane ring bridging the carbons C-3, C-22 and C-4 in ring A, and an acetoxy group at C-11 instead of C-12 in ring C (Figure 2). All ethyl acetate extracts, as well as the isolated new scalarane, showed antibacterial activity against the Gram-positive bacteria *Arthrobacter crystallopoietes* (DSM 20117) and *Bacillus megaterium* (DSM 32), in a screening approach.

## Results

### Chemical investigation on *Doriprismatica stellata* nudibranchs, egg ribbons and *Spongia* cf. *agaricina*

The new molecule was isolated as a white amorphous solid from *D. stellata* nudibranchs (11 mg, 0.3% wet weight). Specific optical rotation was measured in chloroform (*c* = 0.6), giving [α]_D_ +40.5. The molecular formula C_29_H_42_O_6_ was established based on ^13^C NMR data and HRAPCIMS measurements, yielding *m/z* 487.3054 [M + H]^+^ (Supporting Information File 1). The double bond equivalent (DBE) was calculated to be nine and together with the ^13^C NMR data, giving evidence for one C–C and two C–O double bonds, thus suggested a structure with six rings. The presence of a hydroxy group and ester functionalities was deduced from characteristic IR absorptions at 3416, 1732 and 1234 cm^−1^ (Supporting Information File 1) [39–40,53].

The planar structure of 12-deacetoxy-4-demethyl-11,24-diacetoxy-3,4-methylenedeoxoscalarin was established by extensive 1D and 2D NMR experiments (^1^H, ^13^C, ^1^H,^1^H-COSY, DEPT, HSQC and HMBC, see Table 1, Figure S8, Supporting Information File 1). The ^13^C NMR spectrum showed 29 resonances attributable to five methyl groups, nine methylene and eight methine moieties (one olefin: C-16 (δ 117.5), and two oxygen bearing groups: C-11 (δ 68.4) and C-19 (δ 98.9)), and seven quaternary carbons, as obvious from a DEPT135 spectrum. The ^1^H NMR spectrum showed unusual upfield resonances, diagnostic for a cyclopropyl ring H_2_-22 (δ −0.06 brt, *J* = 4.8 Hz, δ 0.43 dd, *J* = 3.9, 9.2 Hz). Furthermore, this spectrum proved the presence of the olefinic proton H-16 (δ 5.49 brs), the downfield shifted methine proton H-11 (δ 5.49 brs), and the hemiacetal hydrogen atom H-19 (δ 5.24 d, *J* = 4.4 Hz). The ^1^H NMR spectrum also featured two downfield shifted methylene systems H_2_-20 (δ 4.44, δ 4.15 d, *J* = 12.2 Hz) and H_2_-24 (δ 4.91, δ 4.81 d, *J* = 12.9 Hz), as well as two acetoxy groups H_3_-11-OAc (δ 2.06 s) and H_3_-24-OAc (δ 2.08 s), and three methyl groups H_3_-21 (δ 0.94 s), H_3_-23 (δ 0.95 s), and H_3_-25 (δ 0.98 s).

**Table 1:** NMR spectroscopic data of 12-deacetoxy-4-demethyl-11,24-diacetoxy-3,4-methylenedeoxoscalarin (CDCl_3_).

C	δ_H_ (mult. *J* in Hz)	δ_C_	COSY	HMBC	NOESY

1β	1.73, m	35.3, CH_2_	H-1α, H-2α/β	C-2, C-3, C-5, C-9, C-10, C-23	H-1α, H-11
1α	0.53, m		H-1β, H-2α/β	C-2, C-3, C-5, C-9, C-10, C-23	H-1β, H-22b
2β	1.96, m	19.0, CH_2_	H-3, H-1α/β, H-2α	C-1, C-2, C-22	H-2α, H-3, H_3_-23
2α	1.70, m		H-3, H-1α/β, H-2β	C-1, C-2, C-22	H-2β
3	0.55, m	17.9, CH	H-22α/β, H-2α/β	C-1, C-2, C-4, C-5, C-10, C-21, C-22	H_3_-21, H_3_-23, H-22a
4		16.1, C			
5	0.92, m	53.2, CH	H-6a/b	C-1, C-6, C-7, C-10, C-21, C-22, C-23	H-9, H-22b
6a	1.68, m	22.1, CH_2_	H-5, H-6b, H-7α/β	C-5, C-7, C-8, C-10	H-6b
6b	1.49, m		H-5, H-6a, H-7α/β	C-5, C-7, C-8, C-10	H-6a
7β	2.42, m	36.5, CH_2_	H-6a/b, H-7α	C-6, C-8, C-9, C-14, C-24	H-7β
7α	0.81, m		H-6a/b, H-7β	C-6, C-8, C-9, C-14, C-24	H-7α, H-14
8		41.2, C			
9	1.01, s	57.4, CH	H-11	C-8, C-10, C-23, C-24	H-5, H-11, H-14
10		36.5, C			
11	5.49, brs	68.4, CH	H-9, H-12α/β	C-8, C-9, C-10, C-11-OAc, C-12, C-13	H-1α, H-9, H-12α/β
12β	2.19, m	44.2, CH_2_	H-11, H-12α	C-11, C-13, C-25	H-12α
12α	1.51, m		H-11, H-12β	C-11, C-13, C-25	H-12β
13		32.5, C			
14	1.40, brt (8.5)	55.0, CH	H-15α/β	C-8, C-9, C-13, C-15, C-18, C-24	H-9, H-18
15a/b	2.27, m	24.0, CH_2_	H-14, H-16		H-16
					
16	5.49, brs	117.5, CH	H-20α/β, H-18, H-15α/β		H-20b, H-15a/b
17		135.6, C			
18	2.15, m	62.7, CH	H-16, H-19	C-13, C-14, C-19, C-25	H-12α, H-14
19	5.24, d (4.4)	98.9, CH	H-18	C-13, C-17, C-18, C-20	H-12β, H_3_-25
20a	4.44, d (12.2)	68.8, CH_2_	H-16, H-20b		H-20b
20b	4.15, d (12.2)		H-16, H-20a	C-16, C-17, C-18, C-19	H-16, H-20a
21	0.94, s	23.3, CH_3_		C-3, C-4, C-5, C-22	H-3, H-22a
22a	0.43, dd (3.9, 9.2)	22.7, CH_2_	H-3, H-22b	C-2, C-5, C-21	H-3, H-22b
22b	-0.06, brt (4.8)		H-3, H-22a	C-2, C-5, C-21	H-1α, H-5, H-22a
23	0.95, s	14.0, CH_3_		C-1, C-5, C-9, C-10	H-3, H-24a
24a	4.91, d (12.9)	64.2, CH_2_	H-24b	C-7, C-8, C-9, C-14, 24-OAc	H_3_-23
24b	4.81, d (12.9)		H-24a	C-7, C-8, C-9, C-14, 24, 24-OAc	H_3_-25
25	0.98, s	16.1, CH_3_		C-12, C-13, C-14, C-18	H-19, H-24b
11-OAc	2.06, s	21.9, CH_3_		C-11	
		170.2, C			
24-OAc	2.08, s	21.3, CH_3_		C-24	
		170.9, C			

^a1^H (600 MHz), ^13^C NMR (150 MHz), all δ in ppm relative to CDCl_3_ = 7.26/77.0. ^b^Multiplicities determined by DEPT.

The analysis of the 2D NMR data and comparison to literature values [53] suggested that the compound belongs to the family of scalarane sesterterpenoids, with similarities to the deoxoscalarin-like molecule 12,24-diacetoxydeoxoscalarin, previously isolated from a Korean sponge of the genus *Spongia* [53]. The two acetoxy groups were located at the C-11 (δ 68.4) and the C-24 (δ 64.2) carbon atoms based on HMBC cross peaks between the methine proton H-11 (δ 5.49 brs) and the carbon atoms C-11-OAc (δ 21.9, 170.2), as well as the methylene protons H_2_-24 (δ 4.91, δ 4.81 d, *J* = 12.9 Hz) and the carbon atoms C-24-OAc (δ 21.3, 170.9). The location of C-24 was apparent from HMBC cross peaks between the methylene protons H_2_-24 (δ 4.91, δ 4.81 d, *J* = 12.9 Hz) and the carbon atoms C-7 (δ 36.5) and C-14 (δ 55.0). The cyclopropyl group was assigned to the C-3 (δ 17.9) and the C-4 (δ 16.1) carbon atoms, based on a ^1^H,^1^H-COSY correlation between the methylene protons H_2_-22 (δ −0.06 brt, *J* = 4.8 Hz, δ 0.43 dd, *J* = 3.9, 9.2 Hz), and the methine proton H-3 (δ 0.55 m), based on HMBC cross peaks between the protons H_2_-22 and the carbon atoms C-2 (δ 19.0), C-5 (δ 53.2) and C-21 (δ 23.3). The entire assignment of all NMR data is given in Table 1.

**Figure 4:**
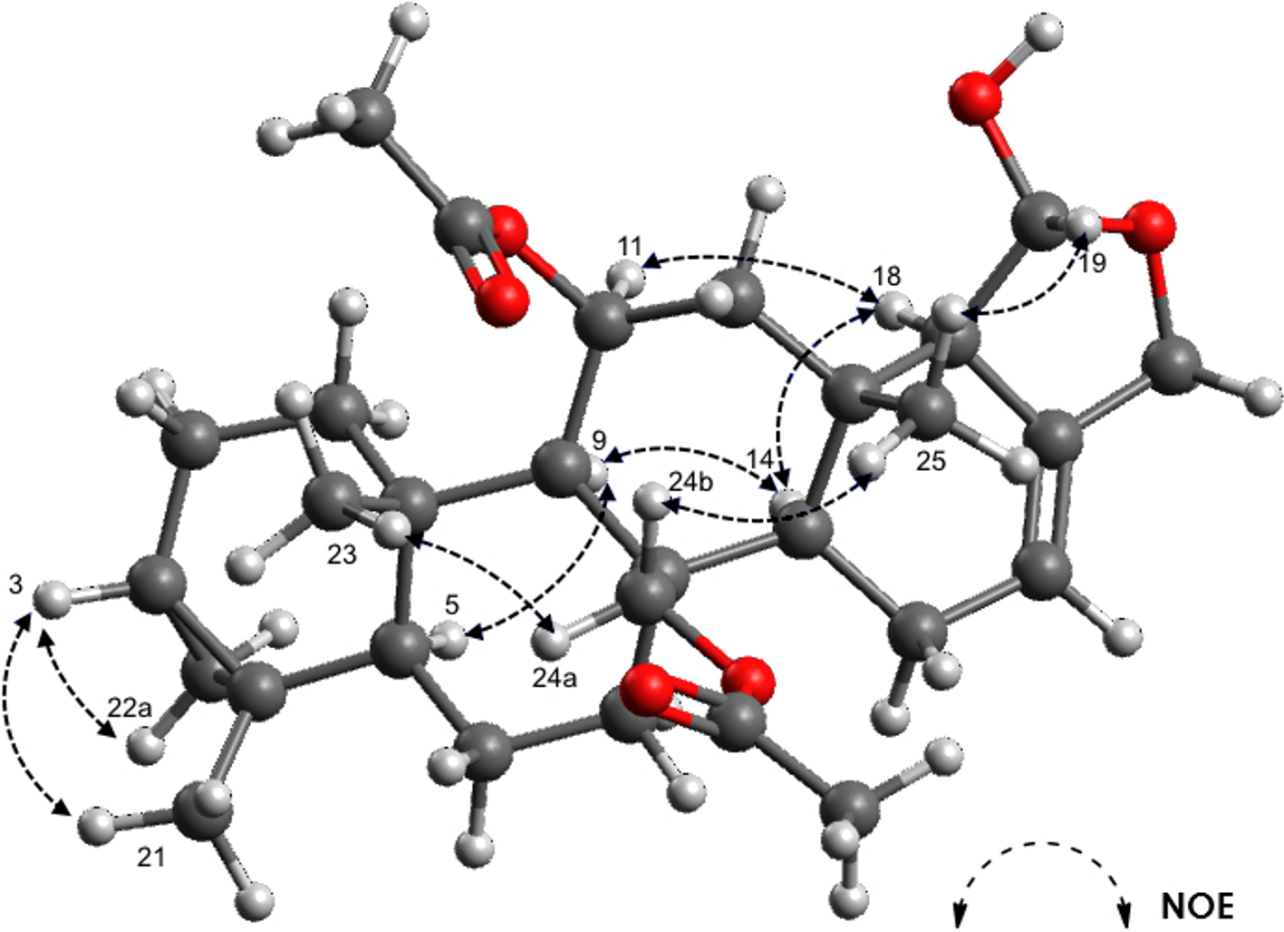
Proposed relative configuration of 12-deacetoxy-4-demethyl-11,24-diacetoxy-3,4-methylenedeoxoscalarin. Selected NOE correlations are indicated with arrows. The model was obtained using Avogadro, an open-source molecular builder and visualization tool, version 1.2.0.

The relative configuration was determined from proton coupling constants and NOE data (Table 1, Figure 4). NOESY cross peaks between H-3 (δ 0.55 m), and H-22a (δ 0.43 dd, *J* = 3.9, 9.2 Hz), H_3_-21 (δ 0.94 s), and H_3_-23 (δ 0.95 s), as well as between H_3_-23 and H-24a (δ 4.91 d, *J* = 12.9 Hz), H-24b (δ 4.81 d, *J* = 12.9 Hz) and H_3_-25 (δ 0.98 s), and between H_3_-25 and H-19 (δ 5.24 d, *J* = 4.4 Hz), indicated that these protons share the same orientation on the molecular plane. The chemical shifts of the angular methyl groups CH_3_-23 (δ 14.0) and CH_3_-25 (δ 16.1) suggested that all ring junctions are *trans* [58–60]. This was supported by NOESY cross peaks between H-22b (δ −0.06 brt, *J* = 4.8 Hz) and H-5 (δ 0.92 m), angular methines H-5 and H-9 (δ 1.01 s), H-9 and H-14 (δ 1.40 brt, *J* = 8.5 Hz), and between H-14 and H-18 (δ 2.15 m), from which a shared α-orientation can be inferred. Moreover, the cross peak between H-19 (δ 5.24 d, *J* = 4.4 Hz) and H_3_-25 (δ 0.98 s), and a coupling constant of *J* = 4.4 Hz between H-19 and H-18, further confirm the *trans* relationship between these protons. Hence, the structure and relative configuration of 12-deacetoxy-4-demethyl-11,24-diacetoxy-3,4-methylenedeoxoscalarin was determined. It needs to be noted that the molecule was unstable over time, especially in ring E, and a variety of degradation products formed by, inter alia, hydrolysis of the hemiacetal and loss of the acetoxy groups.

The new scalarane was also detected in *Doriprismatica stellata* egg ribbons and *Spongia* cf. *agaricina* (Figure 3). It was isolated from both samples (egg ribbons: 1 mg, 0.1% wet weight; sponge: 0.7 mg, 0.02% wet weight) and the identity was validated by comparison of the MS and NMR spectra.

### Antibacterial activity

All ethyl acetate extracts from *Doriprismatica stellata* nudibranchs, egg ribbons and *Spongia* cf. *agaricina* showed antibacterial activity against the Gram-positive *Arthrobacter crystallopoietes* (DSM 20117) in a first screening approach. The pure compound 12-deacetoxy-4-demethyl-11,24-diacetoxy-3,4-methylenedeoxoscalarin, isolated from all three extracts, was active against the Gram-positive *Bacillus megaterium* (DSM 32) (Supporting Information File 1).

## Discussion

In this study, the new scalarane-type sesterterpene 12-deacetoxy-4-demethyl-11,24-diacetoxy-3,4-methylenedeoxoscalarin was isolated from *Doriprismatica stellata* nudibranchs (Gastropoda, Mollusca), their egg ribbons, and the associated sponge *Spongia* cf. *agaricina* (Demospongiae, Porifera), collected from Bunaken National Park (BNP, North Sulawesi, Indonesia). Nudibranchs and their egg ribbons revealed higher concentrations of the scalarane in comparison to the sponge, likely due to a continuous accumulation of this compound.

In general, scalarane sesterterpenes are bioactive metabolites, mainly isolated from marine sources, such as Dictyoceratida sponges and the nudibranchs that feed on them [7,25,29,33,56]. So far, only six scalaranes containing cyclopropane rings, constructed of C-4, C-19 and C-20, have been identified [61–62]. The new 12-deacetoxy-4-demethyl-11,24-diacetoxy-3,4-methylenedeoxoscalarin shared high similarities with 12,24-diacetoxydeoxoscalarin, a farnesoid X-activated receptor antagonist, isolated by Nam et al. from a Korean sponge of the genus *Spongia* [53]. However, differing from the previously reported scalaranes [25,29,33,42,56,61–62], the new metabolite is functionalized at C-11 instead of C-12 and has a cyclopropane ring bridging C-3, C-22 and C-4 of ring A.

Scalarane sesterterpenes are considered as chemotaxonomic markers for the sponge families Thorectidae, Dysideidae and Spongiidae [63–64]. In Spongiidae, they have been isolated from the genera *Coscinoderma* [65], *Hyattella* [52,66], and *Spongia* [53,67–71]. Our results further support this chemotaxonomic classification, by the presence of 12-deacetoxy-4-demethyl-11,24-diacetoxy-3,4-methylenedeoxoscalarin within *Spongia* cf. *agaricina*, (Spongiidae, Dictyoceratida).

Primordially, chromodorid nudibranchs feed upon a broad range of sponges, however, more derived genera like *Glossodoris* and *Doriprismatica* have taken to feeding upon a narrow range of sponges [23,27,47]. As the first chemical investigation of *D. stellata* nudibranchs, our results indicate that these sea slugs live and feed upon the dictyoceratid sponge *Spongia* cf. *agaricina*. This, among other investigations on *Doriprismatica atromarginata* [38,41,44–45,50] and *D. sedna* [39], supports the idea of a stenophagous dietary relationship between nudibranchs of the genus *Doriprismatica* and scalarane-containing dictyoceratid sponges of the families Thorectidae and Spongiidae. This relationship is further reflected by their shared specialized metabolite 12-deacetoxy-4-demethyl-11,24-diacetoxy-3,4-methylenedeoxoscalarin, as proven in this study. Sesterterpenes are a rare terpene class, accounting for less than 2% of all known terpenoids, with only a few reports on their biosynthesis [72–76]. However, their frequent occurrence in marine organisms is striking and sponges are considered as the prime source of these terpenoids [25]. Yet determining the origin and in vitro production of these metabolites is anything but trivial. Sponges are known to host complex symbiont communities, with up to 30–60% as microbial biomass [13,77]. These highly species-specific communities are most probably vertically transmitted [78] and were shown to share and cover various core functions of sponge metabolism by functionally equivalent symbionts, analogous enzymes, or biosynthetic pathways [16,79–80]. Another *Spongia* species, *S. officinalis*, was shown to harbour bacteria with terpenoid cyclases/protein prenyltransferases responsible for a wide chemodiversity of terpenoid natural products [14,81]. Besides, the marine fungi *Penicillium* spp. and *Aspergillus* spp. are often associated with sponge hosts and were found to produce various terpenoids as well [15,82–83]. Hence, if sponges are not the origin of these metabolites, it is tempting to argue that the sesterterpene biosynthesis could be performed or mediated by their microbial symbionts. This further indicates a close association, interconnectedness, and probable co-evolution between microorganisms, sponges and nudibranchs [9]. *D. stellata* was not only found to sequester and accumulate 12-deacetoxy-4-demethyl-11,24-diacetoxy-3,4-methylenedeoxoscalarin from *Spongia* cf. *agaricina*, but to pass it on to the egg ribbons as well. This, in addition to its bioactivity, might suggest a biological role, either as protection against predation, fouling, or in the reproductive cycle, as mentioned in previous studies on nudibranch egg ribbons [17,34–36]. The antibacterial activity of 12-deacetoxy-4-demethyl-11,24-diacetoxy-3,4-methylenedeoxoscalarin could point towards a potential protective role against bacterial biofilm formation. Unfortunately, the metabolite was unstable over time and it was not possible to conduct further assays. Future studies on scalarane sesterterpenes could reveal their full potential and true biological and ecological functions in complex, co-evolved communities.

## Experimental

### General experimental procedures

Optical rotations were measured with a Jasco DIP 140 polarimeter. UV and IR spectra were obtained using Perkin-Elmer Lambda 40 and Perkin-Elmer Spectrum BX instruments, respectively. All NMR spectra were acquired in base-filtered CDCl_3_ using Bruker Avance 300 DPX or Bruker Ascend 600 with prodigy cryoprobe spectrometers. Spectra were referenced to residual solvent signals with resonances at δ_H/C_ 7.26/77.00 ppm (CDCl_3_). Mass spectra were recorded on a micrOTOF-Q mass spectrometer (Bruker) with ESI-source coupled with an HPLC Dionex Ultimate 3000 (Thermo Scientific) using an Agilent Zorbax Eclipse Plus C_18_ column (2.1 × 50 mm, 1.8 µm) at a temperature of 45 °C. MS data were acquired over a range from 100–3000 *m/z* in positive mode. Auto MS/MS fragmentation was achieved with rising collision energy (35–50 keV over a gradient from 500–2000 *m/z*) with a frequency of 4 Hz for all ions over a threshold of 100. UHPLC started with 90% H_2_O containing 0.1% acetic acid. The gradient began after 0.5 min to 100% acetonitrile (0.1% acetic acid) in 4 min. 2 µL of a 1 mg/mL sample solution was injected to a flow of 0.8 mL/min. HRAPCIMS were recorded on LTQ Orbitrap XL mass spectrometer. HPLC was carried out on a Waters Breeze HPLC system equipped with a 1525µ dual pump, a 2998 DAD detector, and a Rheodyne 7725i injection system and with a Waters Alliance HPLC system equipped with a Waters 2695 separation module and a Waters 996 PDA detector. A Macherey-Nagel Nucleodur C_18_ Pyramid column (250 mm × 10 mm; 5 µm) and a Phenomenex Kinetex C_18_ column (250 mm × 4.6 mm, 5 µm) were used for separation.

### Biological material

Samples of *Doriprismatica stellata* sea slugs (Nudibranchia, Gastropoda, Mollusca), their egg ribbons and pieces of the sponge, on which they were found (1.2 g, 0.7 g, and 3.5 g wet weight, respectively) were collected via scuba diving in August 2015 during a field trip to Bunaken National Park (BNP, North Sulawesi, Indonesia, 1° 37' 51'' N, 124° 45' 05'' E) at the coral reef drop off. Four additional *D. stellata* sea slugs (2.5 g wet weight) were collected in October 2016 during another field trip to BNP. The nudibranchs and associated egg ribbons were identified as *Doriprismatica stellata* by H. Wägele and N. Undap at the Zoological Research Museum Alexander Koenig, Bonn, Germany [84–85]. The sponge displayed a foliose habit with brownish-violett pigmentation and was identified as *Spongia* cf. *agaricina* using methods as described by Ackers et al. in 2007 [86], see also Erpenbeck et al. from 2020 [87] (Supporting Information File 1). Specimens were stored in ethanol (96%) at −20 °C until further extraction and processing in the laboratories at the University of Bonn. A part of the collected sea slug and substrate materials will be finally stored at the Sam Ratulangi University, Manado, Indonesia, in the Reference Collection under the numbers SRU2015/01 and SRU2016/02. A fraction of the sponge material is stored in the Bavarian State Collection for Paleontology and Geology under collection number SNSB-BSPG.GW41291.

### Extraction and isolation

Six *Doriprismatica stellata* nudibranchs (3.7 g wet weight), their egg ribbons (0.7 g wet weight) and pieces of the associated sponge (3.5 g wet weight) were separately frozen, crushed and ultrasonicated for a total of 3 minutes (30 s intervals) on ice, while submerged in a minimum of first acetone (Ac) and consecutively methanol (MeOH). The ethanolic storage solutions of *D. stellata* nudibranch, egg ribbon, and *Spongia* cf. *agaricina* samples were each combined with the respective Ac/MeOH extracts of the samples and dried under vacuum to give the crude extracts. After liquid–liquid separation of the three crude extracts (0.9 g, 0.3 g, and 0.2 g, respectively) between 50 mL water (H_2_O) and three times 50 mL ethyl acetate (EtOAc), EtOAc solubles (223 mg, 35 mg, and 81 mg) were separated by RP-HPLC. A Phenomenex Kinetex C_18_ column (250 mm × 4.6 mm, 5 µm), with a linear gradient elution from 70:30 (MeOH/H_2_O) to 100% MeOH in 25 min, and a flow of 1.5 mL/min was used for separation. The isolated metabolite had a retention time around 13 minutes.

### 12-Deacetoxy-4-demethyl-11,24-diacetoxy-3,4-methylenedeoxoscalarin

C_29_H_42_O_6,_ white amorphous solid (12.7 mg); [α]_D_^20^ +40.5 (*c* 0.6, CHCl_3_); IR (ATR) *v*_max_: 3416, 2922, 2861, 1732, 1234 cm^−1^; ^1^H and ^13^C NMR (Table 1); HRAPCIMS (*m/z*): [M + H]^+^ calcd. for C_29_H_43_O_6_, 487.3060; found, 487.3054.

## Supporting Information

File 1 Spectroscopic data and other relevant information for 12-deacetoxy-4-demethyl-11,24-diacetoxy-3,4-methylenedeoxoscalarin.
