# Supplementary material for: Antibacterial scalarane from Doriprismatica stellata nudibranchs (Gastropoda, Nudibranchia), egg ribbons, and their dietary sponge Spongia cf. agaricina (Demospongiae, Dictyoceratida)
Source: Beilstein J Org Chem. 2020 Jul 3;16:1596–605. doi: 10.3762/bjoc.16.132 (PMC7356558; doi:10.3762/bjoc.16.132)
Supplement: File 1 — Spectroscopic data and other relevant information for 12-deacetoxy-4-demethyl-11,24-diacetoxy-3,4-methylenedeoxoscalarin. [file Beilstein_J_Org_Chem-16-1596-s001.pdf]

BEILSTEIN JOURNAL OF ORGANIC CHEMISTRY

Supporting Information

for

Antibacterial scalarane from *Doriprismatica stellata* nudibranchs (Gastropoda, Nudibranchia), egg ribbons, and their dietary sponge *Spongia* cf. *agaricina* (Demospongiae, Dictyoceratida)

Cora Hertzer, Stefan Kehraus, Nils Böhringer, Fontje Kaligis, Robert Bara,
Dirk Erpenbeck, Gert Wörheide, Till F. Schäberle, Heike Wägele and Gabriele M. König

Beilstein J. Org. Chem. **2020,** *16,* 1596–1605. doi:10.3762/bjoc.16.132

Spectroscopic data and other relevant information for 12-deacetoxy-4-demethyl-11,24-diacetoxy-3,4-methylenedeoxoscalarin

License and Terms: This is a supporting information file under the terms of the Creative Commons Attribution License (http://creativecommons.org/licenses/by/4.0). Please note that the reuse, redistribution and reproduction in particular requires that the authors and source are credited.
The license is subject to the *Beilstein Journal of Organic Chemistry* terms and conditions: (https://www.beilstein-journals.org/bjoc)

Table of contents

Figure S1. ^{1}H NMR spectrum of 12-deacetoxy-4-demethyl-11,24-diacetoxy-3,4-methylenedeoxoscalarin in $CDCl_3$. S2
Figure S2. ^{13}C NMR spectrum of 12-deacetoxy-4-demethyl-11,24-diacetoxy-3,4-methylenedeoxoscalarin in $CDCl_3$. S2
Figure S3. DEPT spectrum of 12-deacetoxy-4-demethyl-11,24-diacetoxy-3,4-methylenedeoxoscalarin in $CDCl_3$. S3
Figure S4. HSQC spectrum of 12-deacetoxy-4-demethyl-11,24-diacetoxy-3,4-methylenedeoxoscalarin in $CDCl_3$. S3
Figure S5. HMBC spectrum of 12-deacetoxy-4-demethyl-11,24-diacetoxy-3,4-methylenedeoxoscalarin in $CDCl_3$. S4
Figure S6. COSY spectrum of 12-deacetoxy-4-demethyl-11,24-diacetoxy-3,4-methylenedeoxoscalarin in $CDCl_3$. S4
Figure S7. NOESY spectrum of 12-deacetoxy-4-demethyl-11,24-diacetoxy-3,4-methylenedeoxoscalarin in $CDCl_3$. S5
Figure S8. Key COSY and HMBC correlations of 12-deacetoxy-4-demethyl-11,24-diacetoxy-3,4-methylenedeoxoscalarin. S5
Figure S9. Key NOE correlations of 12-deacetoxy-4-demethyl-11,24-diacetoxy-3,4-methylenedeoxoscalarin. S6
Figure S10. UV spectrum of 12-deacetoxy-4-demethyl-11,24-diacetoxy-3,4-methylenedeoxoscalarin in ACN. S6
Figure S11. IR (ATR) spectrum of 12-deacetoxy-4-demethyl-11,24-diacetoxy-3,4-methylenedeoxoscalarin. S7
Figure S12. HRAPCIMS measurement of 12-deacetoxy-4-demethyl-11,24-diacetoxy-3,4-methylenedeoxoscalarin. S7
Figure S 13 Reticulate spongin fibre skeletal arrangement of *S.* cf. *agaricima* S8
Figure S14 Antibacterial activity assay of extracts and the pure compound 12-deacetoxy-4-demethyl-11,24-diacetoxy-3,4-methylenedeoxoscalarin against Gram-positive bacteria S11

Table S1. Recipe for 53. Corynebacterium liquid medium S8
Table S2. Raw data OD measurements of antibacterial activity assays. S9

Figure S1: ^{1}H NMR spectrum of 12-deacetoxy-4-demethyl-11,24-diacetoxy-3,4-methylenedeoxoscalarin in $CDCl_3$.

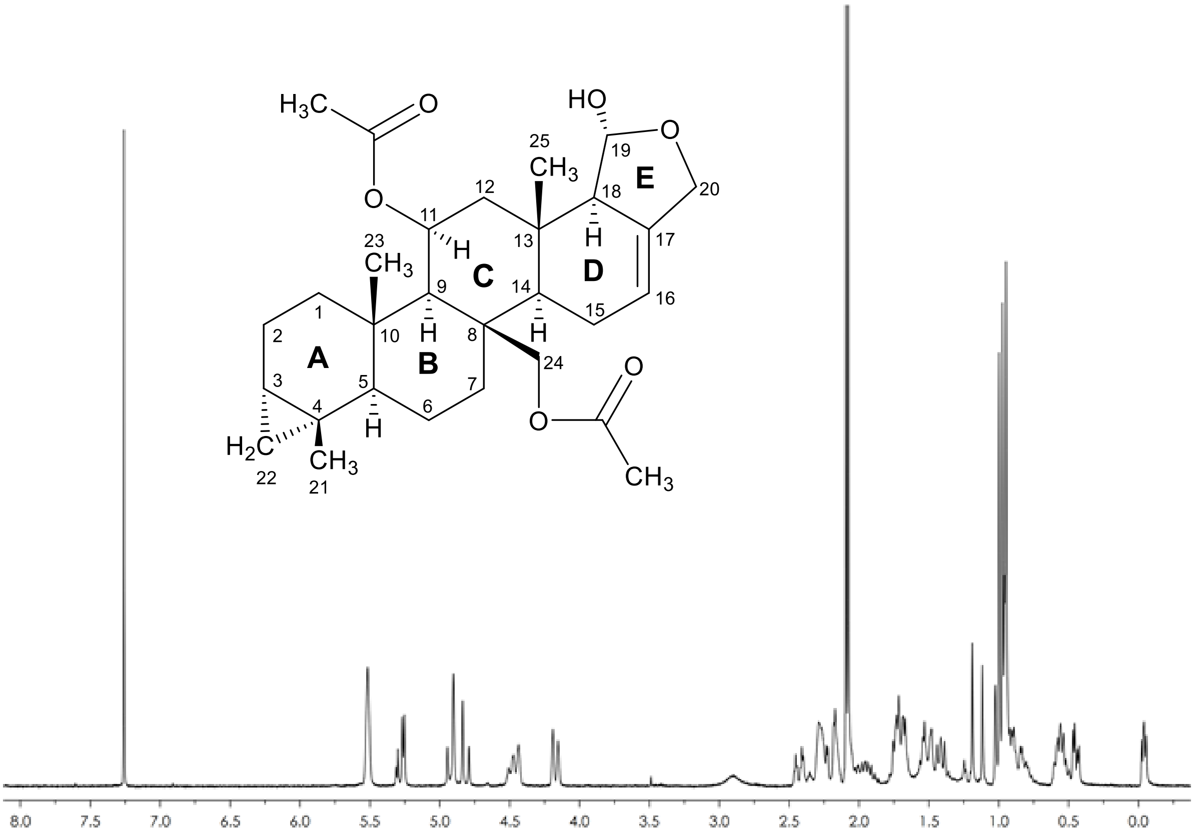

Figure S2: ^{13}C NMR spectrum of 12-deacetoxy-4-demethyl-11,24-diacetoxy-3,4-methylenedeoxoscalarin in $CDCl_3$.

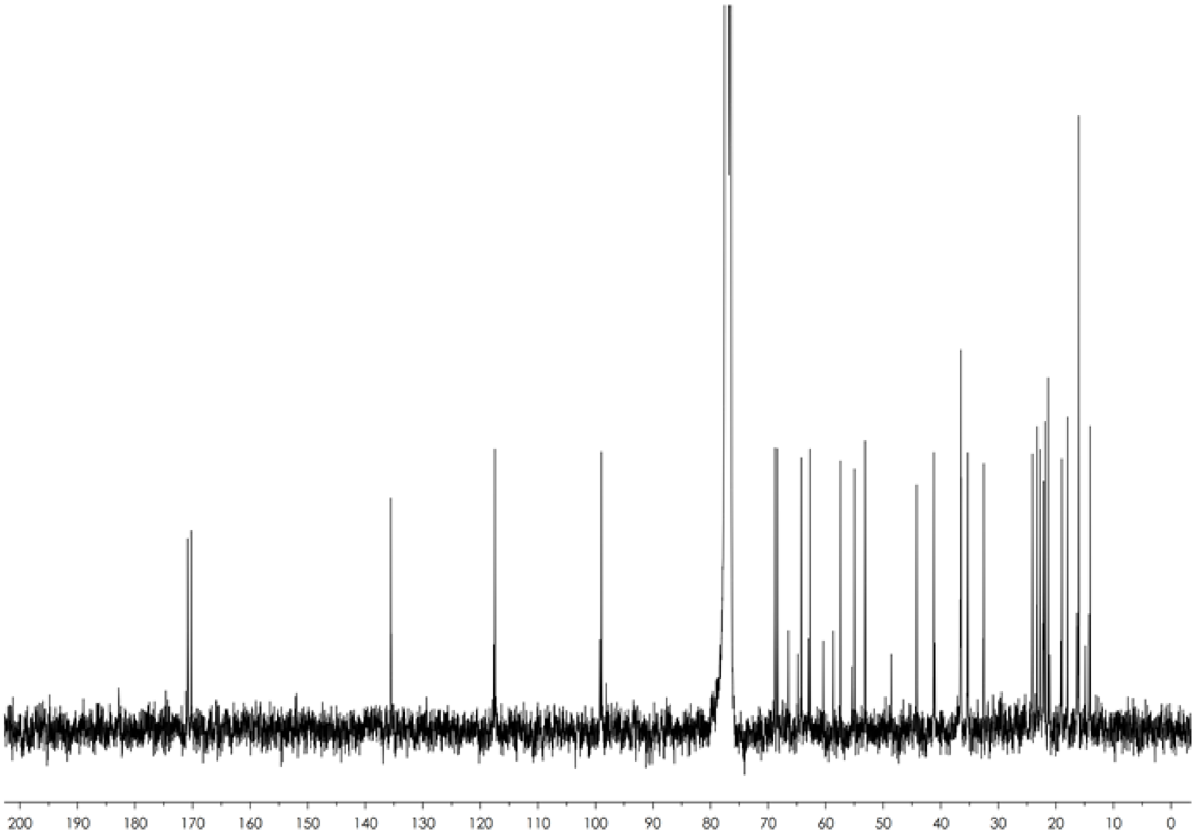

Figure S3: DEPT spectrum of 12-deacetoxy-4-demethyl-11,24-diacetoxy-3,4-methylenedeoxoscalarin in $CDCl_3$.

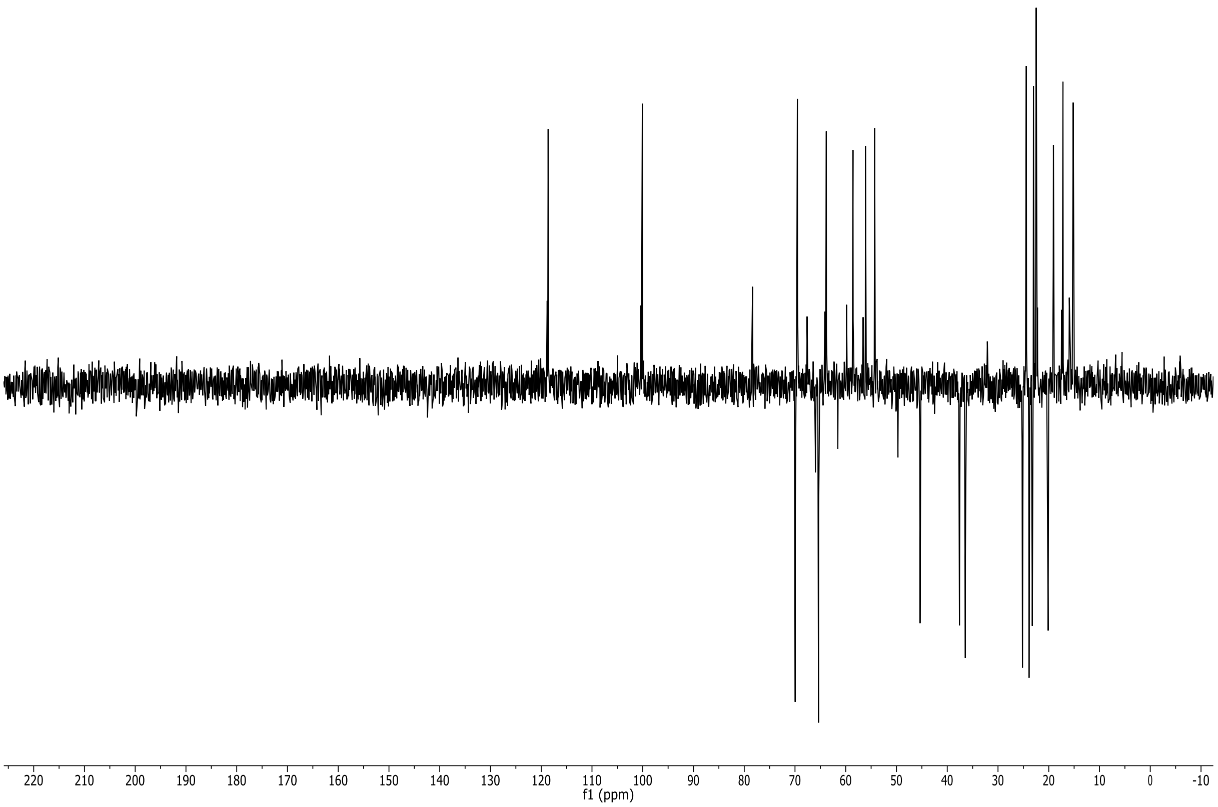

Figure S4: HSQC spectrum of 12-deacetoxy-4-demethyl-11,24-diacetoxy-3,4-methylenedeoxoscalarin in $CDCl_3$.

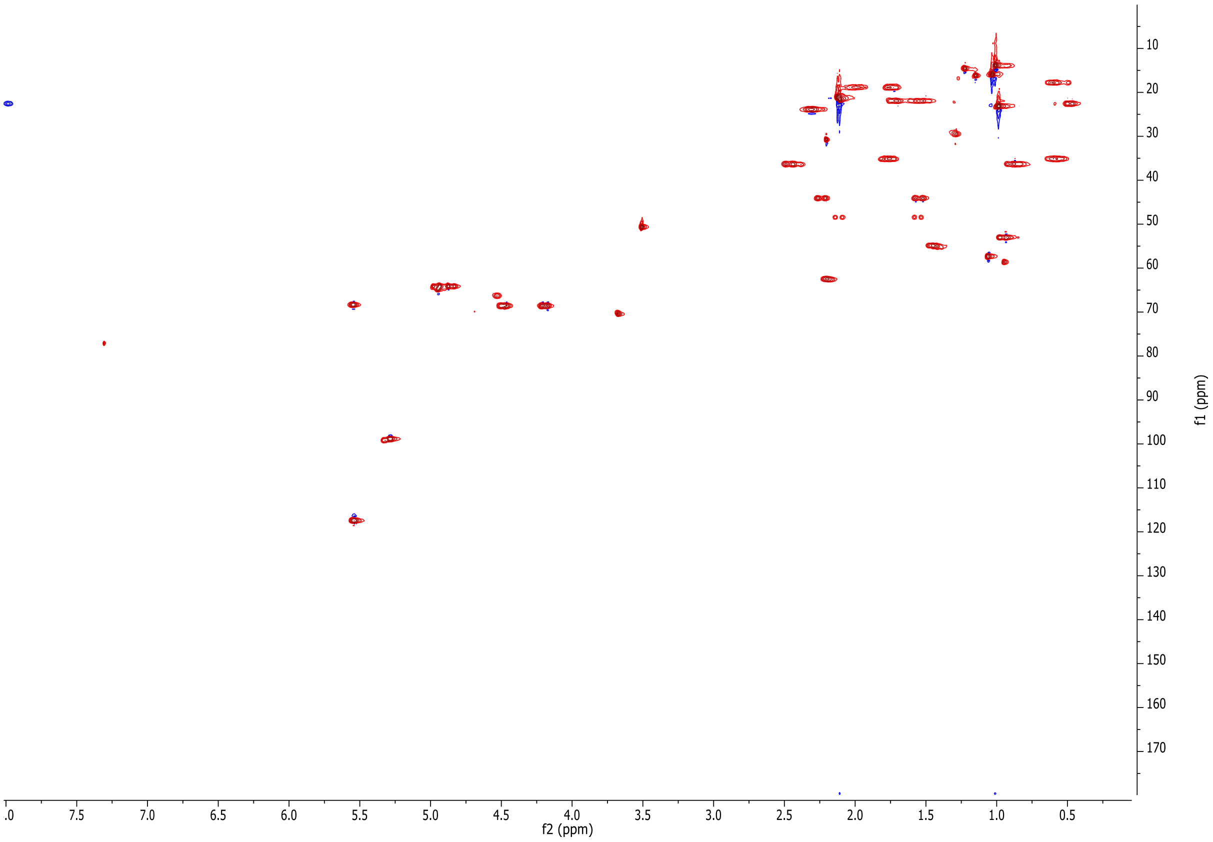

Figure S5: HMBC spectrum of 12-deacetoxy-4-demethyl-11,24-diacetoxy-3,4-methylenedeoxoscalarin in $CDCl_3$.

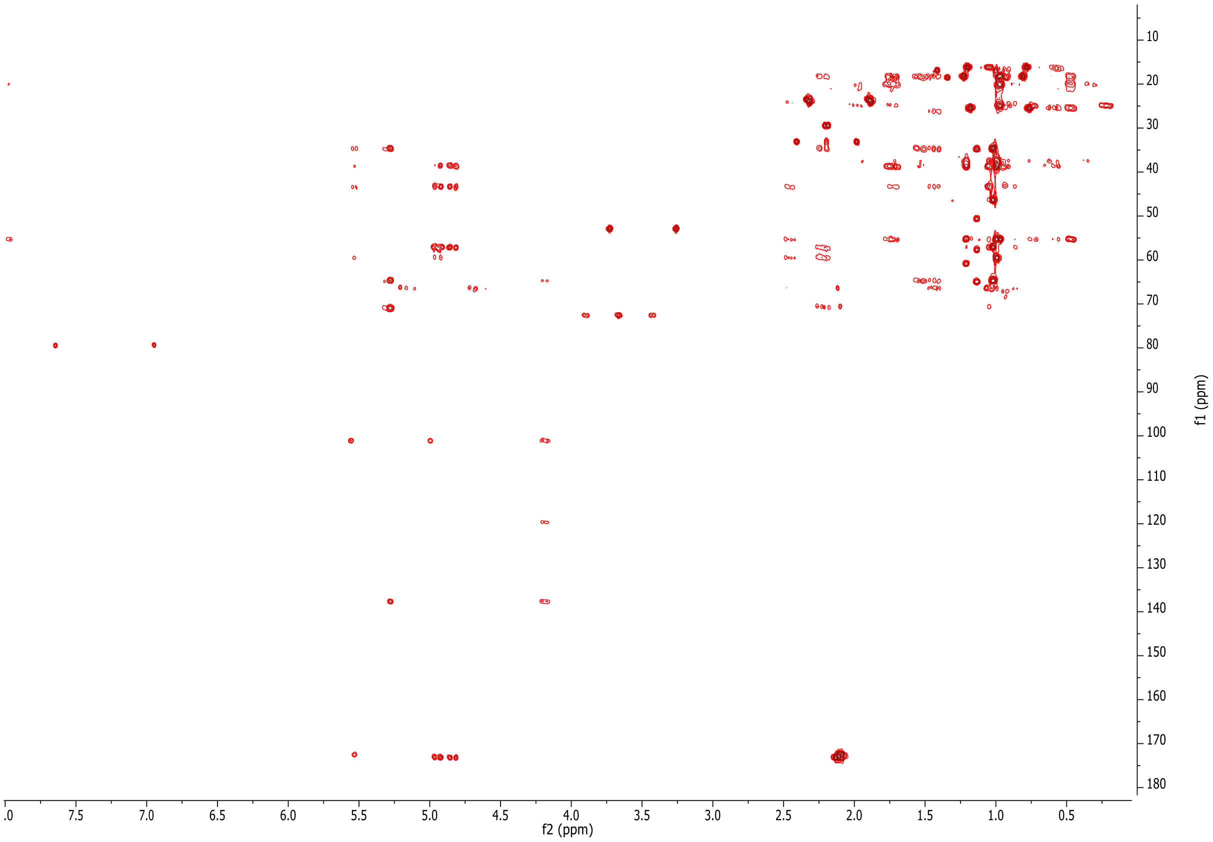

Figure S6: COSY spectrum of 12-deacetoxy-4-demethyl-11,24-diacetoxy-3,4-methylenedeoxoscalarin in $CDCl_3$.

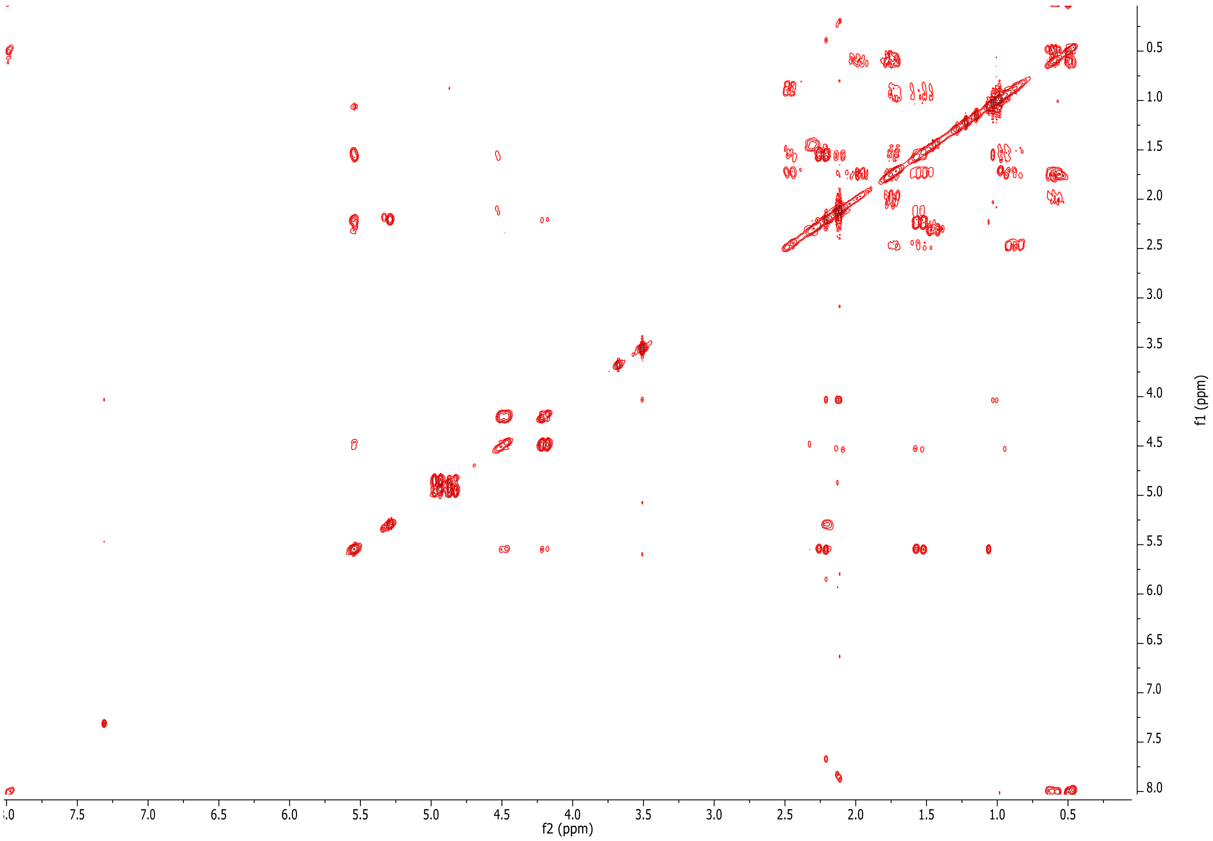

Figure S7: NOESY spectrum of 12-deacetoxy-4-demethyl-11,24-diacetoxy-3,4-methylenedeoxoscalarin in $CDCl_3$.

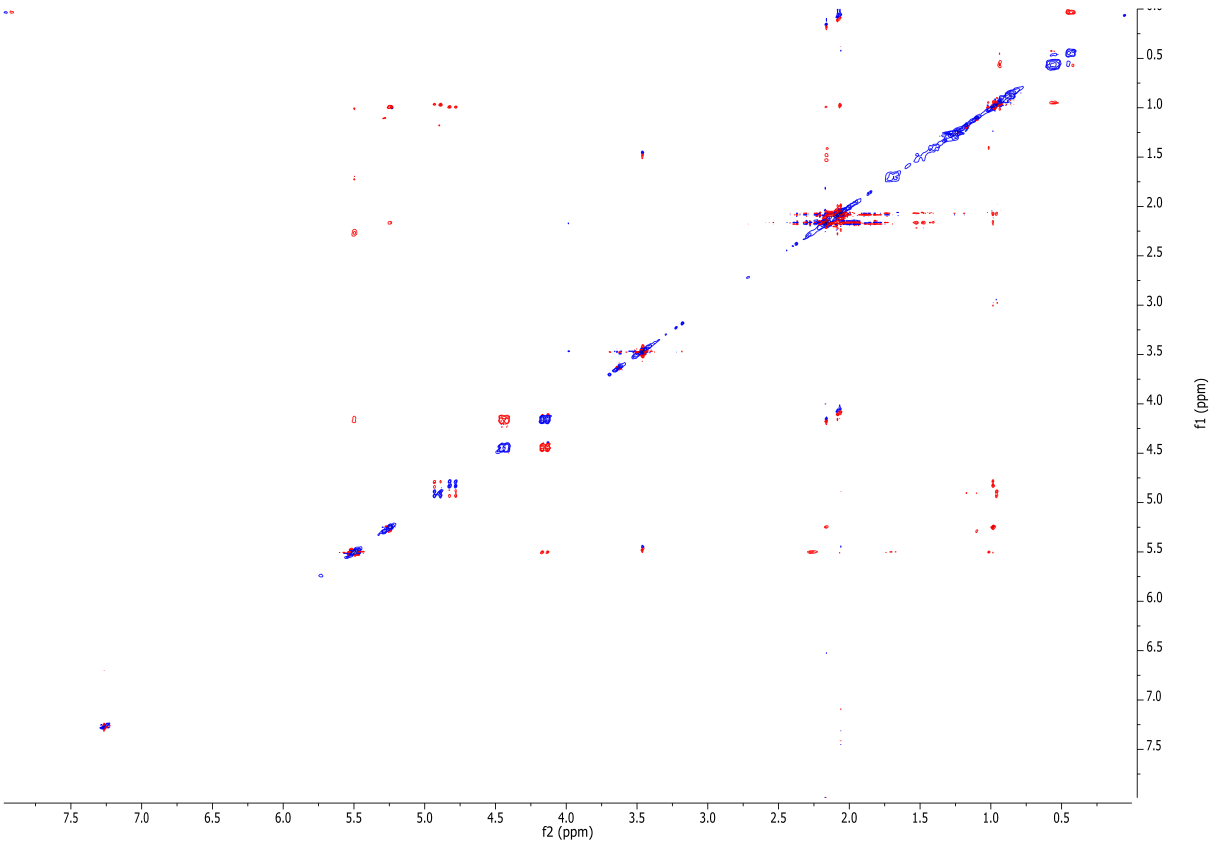

Figure S8: Key COSY and HMBC correlations of 12-deacetoxy-4-demethyl-11,24-diacetoxy-3,4-methylenedeoxoscalarin.

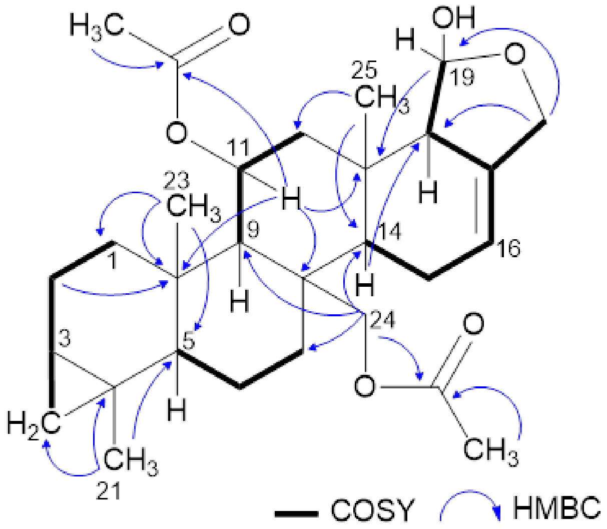

Figure S9: Key NOE correlations of 12-deacetoxy-4-demethyl-11,24-diacetoxy-3,4-methylenedeoxoscalarin.

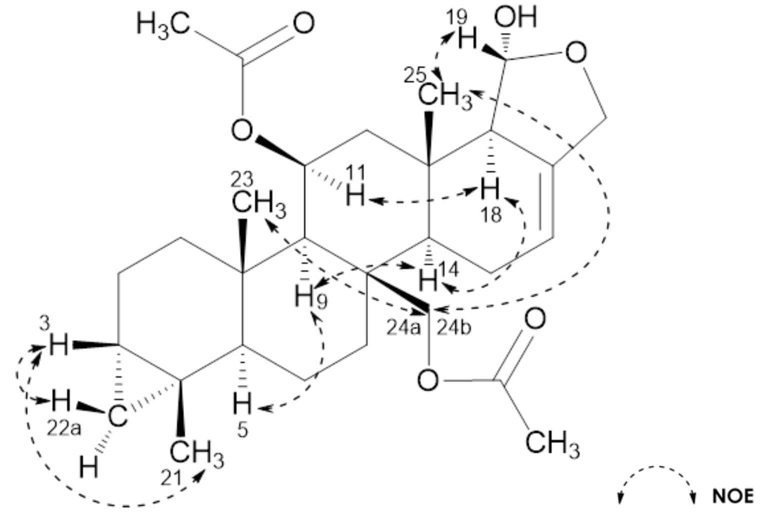

Figure S10: UV spectrum of 12-deacetoxy-4-demethyl-11,24-diacetoxy-3,4-methylenedeoxoscalarin in ACN.

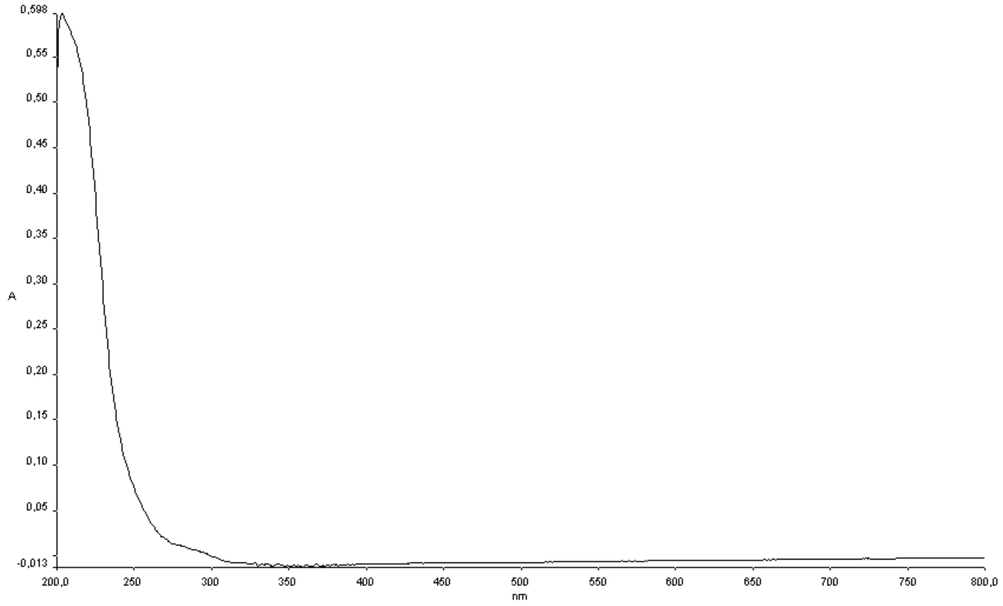

Figure S11: IR (ATR) spectrum of 12-deacetoxy-4-demethyl-11,24-diacetoxy-3,4-methylenedeoxoscalarin.

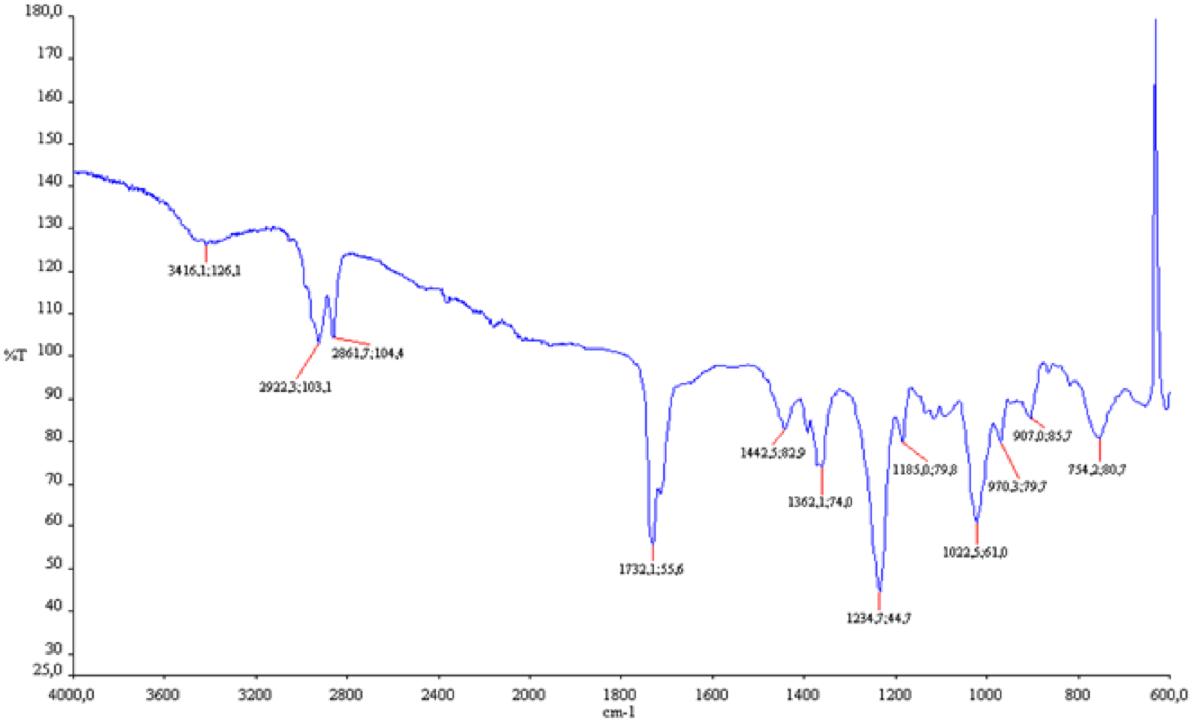

Figure S12: HRAPCIMS measurement of 12-deacetoxy-4-demethyl-11,24-diacetoxy-3,4-methylenedeoxoscalarin, yielding *m/z* 487.3054 [M + H]$^+$, calcd. 487.3060.

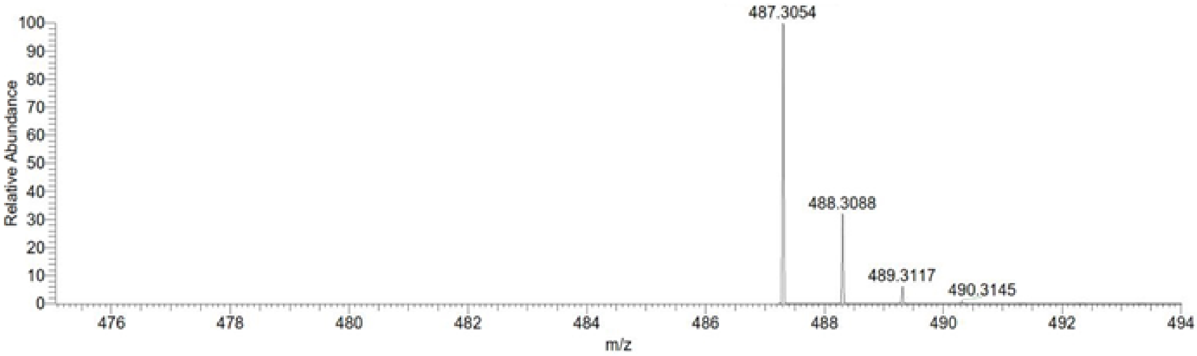

Figure S13: Reticulate spongin fibre skeletal arrangement of *S*. cf. *agaricima*

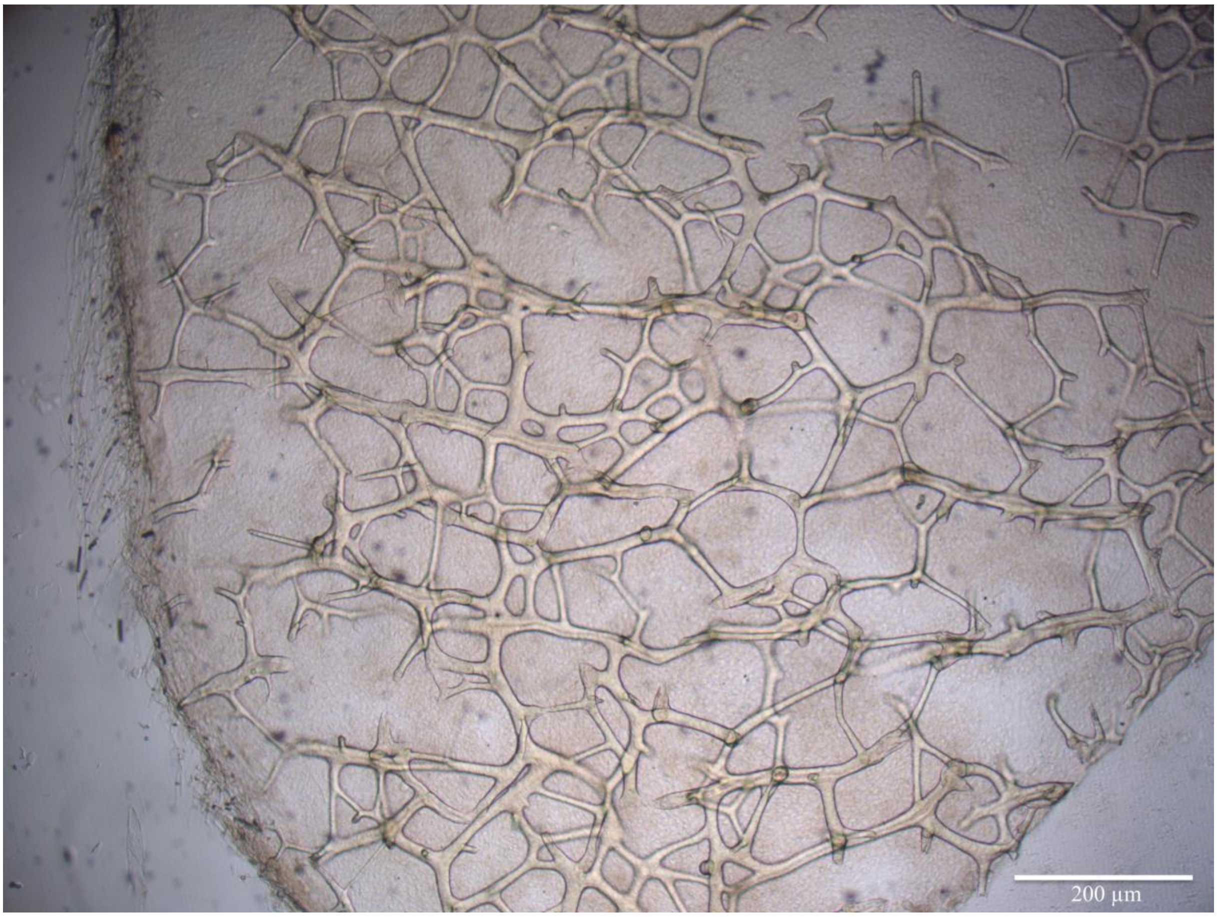

Table S1: Recipe for 53. Corynebacterium liquid medium

Casein peptone, tryptic digest	10 g
Yeast extract	5 g
Glucose	5 g
NaCl	5 g
Distilled water	1000 mL

Adjust pH to 7.2–7.4

Table S2: Raw data OD measurements of antibacterial activity assays.

EtOAc-fractions of *D. stellata* nudibranchs, egg ribbons and the dietary sponge *Spongia* cf. *agaricina* tested against *Arthrobacter crystallopoietes* (DSM 20117). Concentrations of 50, 100 and 200 µg/mL per well, respectively, were used. Antibacterial activity was measured as bacterial growth inhibition in liquid media. Mean OD values (λ 560 nm) of negative DMSO controls were set as 100% (maximal bacteria growth and OD). Percentages for individual sample wells were calculated as individual sample OD*100/mean OD negative DMSO controls. Sample values marked in blue.

SUNRISE; Serial number: 605000077; Firmware: V 3.31 25/08/05; XFLUOR4 Version: V 4.51

Date:	25.1.16
Time:	15:38
Measurement mode:	Absorbance
Measurement wavelength:	560 nm
Read mode:	Center

Rawdata

<>												
A	1,0980	0,9630	0,1780	0,1070	0,1090	0,1100	0,1330	0,1890	0,1800	1,0970	1,2330	1,1000
B	1,1370	0,9620	0,9870	0,1360	1,3260	1,4470	0,3050	0,1420	0,1380	1,2430	1,1960	1,2100
C	0,8790	0,9660	1,0300	0,9270	1,1040	1,1640	0,3620	0,2880	0,2180	1,2630	1,1710	1,0580
D	0,9750	1,0450	1,0330	0,1280	0,2420	0,2980	0,2854	0,2000	0,1930	1,2180	1,2680	1,1360
E	0,1380	0,9210	0,8040	0,2070	1,2720	1,1440	1,3250	1,2970	0,9140	1,2780	1,2620	1,2680
F	0,9490	0,9830	1,0000	0,2160	1,1080	1,1380	0,9910	1,0190	0,9070	1,2760	1,1750	1,1450
G	0,1840	0,2110	0,3920	1,1270	0,9280	1,0510	0,9750	1,0780	1,2420	0,1500	0,1650	0,1390
H	0,1170	0,1240	0,1200	0,1680	1,1360	1,1940	1,2770	1,2920	1,2800	1,0570	0,8860	1,3270

Calculated percentages of *Arthrobacter crystallopoietes* (DSM 20117) bacterial growth in comparison to the negative DMSO controls:

	**50 µg/mL**	**100 µg/mL**	**200 µg/mL**
D. stellata nudibranchs	28,0 %	13,0 %	12,7 %
D. stellata egg ribbons	33,2 %	26,4 %	20,0 %
S. cf. *agaricina*	26,2 %	18,3 %	17,7 %
(+) Control Carbenicillin	15,1 %	13,8 %	12,8 %

After complete structure elucidation the isolated new scalarane 12-deacetoxy-4-demethyl-11,24-diacetoxy-3,4-methylenedeoxoscalarin was tested against *Bacillus megaterium* (DSM 32). Concentrations of 100 µg/mL (205 µM), 50 µg/mL (103 µM) and 25 µg/mL (51 µM) per well, respectively, were used. Sample values marked in blue.

SUNRISE; Serial number: 605000077; Firmware: V 3.31 25/08/05; XFLUOR4 Version: V 4.51

Date:	12.5.17
Time:	10:39
Measurement mode:	Absorbance
Measurement wavelength:	560 nm
Read mode:	Center

Rawdata

<>												
A	0,9180	0,7280	0,7790	1,1300	0,5990	0,7090	0,8250	0,6990	0,7560	0,7370	0,8910	0,8870
B	0,6060	0,7630	0,8890	0,8690	0,6480	0,7100	1,0170	0,6910	0,7520	0,7330	0,6500	0,9720
C	1,0320	0,6760	0,7790	0,7090	0,5460	0,5600	0,7590	0,7360	0,6200	0,5920	0,7860	1,0220
D	0,6220	0,8480	0,5930	0,6950	1,4880	1,0860	0,9800	0,7400	0,6990	0,7230	0,6230	0,9240
E	1,0280	0,9770	0,7980	0,7550	0,7930	0,9210	0,8050	0,7770	0,6840	0,7660	1,1460	0,8380
F	0,6140	0,5580	0,5580	0,6640	1,0300	1,3960	1,5200	1,2160	0,0610	0,0030	0,1040	0,1650
G	0,6940	0,8100	0,9600	1,1440	0,9830	1,0780	1,3600	1,0600	1,1430	1,0820	1,2730	1,1320
H	0,1470	0,1450	0,1520	0,0960	0,1400	0,2160	0,6570	1,0870	1,3260	1,1350	1,0830	1,1520
MW	0,7663	0,7707	0,9477	1,1803	0,7663	0,7707	0,9477	1,1803	0,7663	0,7707	0,9477	1,1803

<>												
A	119,79	94,46	82,20	95,74	78,16	92,00	87,06	59,22	98,65	95,63	94,02	75,15
B	79,08	99,01	93,81	73,62	84,56	92,13	107,32	58,54	98,13	95,11	68,59	82,35
C	134,67	87,72	82,20	60,07	71,25	72,66	80,09	62,36	80,90	76,82	82,94	86,59
D	81,17	110,03	62,57	58,88	194,17	140,92	103,41	62,69	91,21	93,81	65,74	78,28
E	134,15	126,77	84,21	63,96	103,48	119,51	84,95	65,83	89,26	99,39	120,93	71,00
F	80,12	72,40	58,88	56,26	134,41	181,14	160,39	103,02	7,96	0,39	10,97	13,98
G	90,56	105,10	101,30	96,92	128,27	139,88	143,51	89,81	149,15	140,40	134,33	95,91
H	5,48	7,14	8,13	9,60	18,27	28,03	69,33	92,09	173,03	147,28	114,28	97,60

Calculated percentages of *Bacillus megaterium* (DSM 32) bacterial growth in comparison to the negative DMSO controls:

	**25 µg/mL**	**50 µg/mL**	**100 µg/mL**
Pure new Scalarane	14,0 %	11,0 %	0,4 %
(+) Control Carbenicillin	9,6 %	8,1 %	7,1 %

Figure S14: Antibacterial activity assay of extracts and the pure compound 12-deacetoxy-4-demethyl-11,24-diacetoxy-3,4-methylenedeoxoscalarin against Gram-positive bacteria

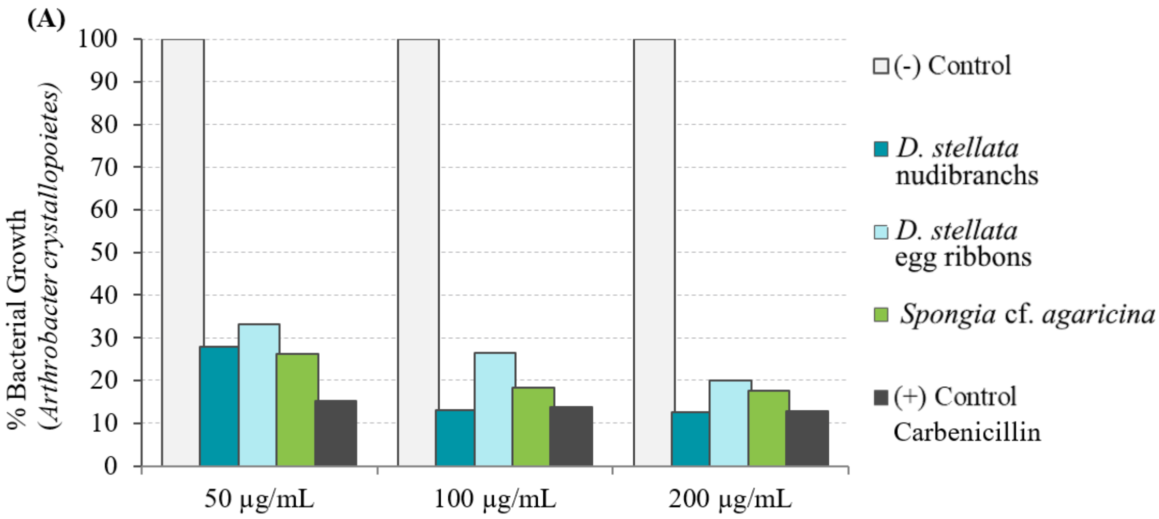

EtOAc-fractions of *D. stellata* nudibranchs, egg ribbons and the dietary sponge *Spongia* cf. *agaricina* tested against *Arthrobacter crystallopoietes* (DSM 20117). The bacterial growth was inhibited and reduced to 28.0% (nudibranchs), 33.2% (eggs) and 26.2% (sponge) at 50 µg/mL, to 13.0% (nudibranchs), 26.4 % (eggs), and 18.3% (sponge) at 100 µg/mL, and to 12.7% (nudibranchs), 20.0% (eggs), and 17.7% (sponge) at 200 µg/mL, in comparison to the negative control (DMSO).

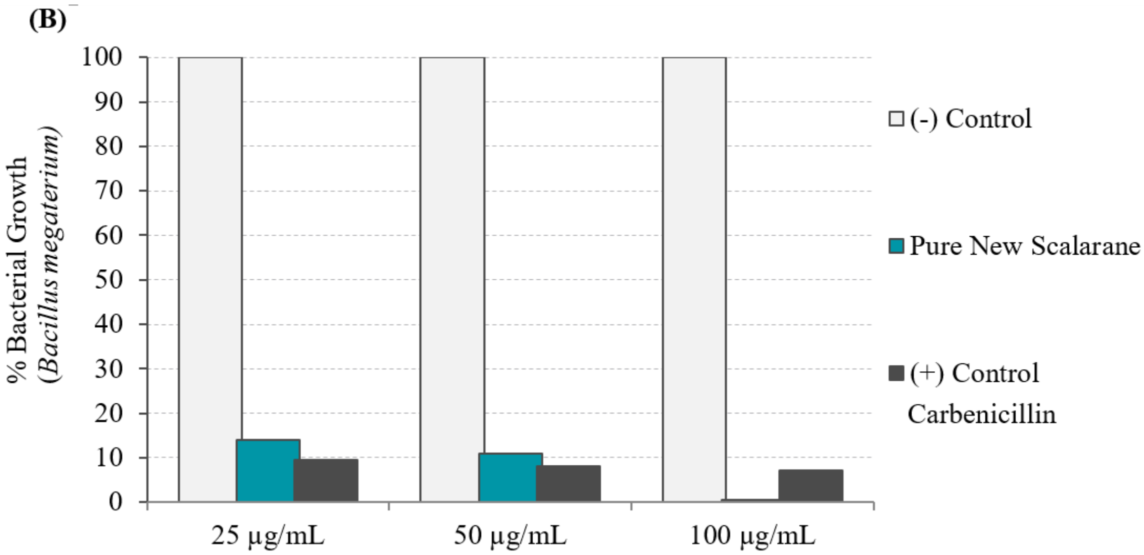

Isolated new scalarane 12-deacetoxy-4-demethyl-11,24-diacetoxy-3,4-methylenedeoxoscalarin tested against *Bacillus megaterium* (DSM 32). The pure scalarane reduced bacterial growth to 0.4% at 100 µg/mL (205 µM), 11.0% at 50 µg/mL (103 µM), and 14.0% at 25 µg/mL (51 µM) in comparison to the negative control (DMSO). Antibacterial activity measured as bacterial growth inhibition in a liquid medium. Mean OD values (λ 560 nm) of negative DMSO controls set as 100% (maximal bacteria growth and OD). Percentages for individual sample wells calculated as individual sample OD*100/mean OD negative DMSO controls.
